# Investigating vibrational relaxation in cyanide-bridged transition metal mixed-valence complexes using two-dimensional infrared and infrared pump-probe spectroscopies

**DOI:** 10.1063/1.4943766

**Published:** 2016-03-15

**Authors:** Karla M. Slenkamp, Michael S. Lynch, Jennifer F. Brookes, Caitlin C. Bannan, Stephanie L. Daifuku, Munira Khalil

**Affiliations:** Department of Chemistry, University of Washington, P.O. Box 351700, Seattle, Washington 98195, USA

## Abstract

Using polarization-selective two-dimensional infrared (2D IR) and infrared pump-probe spectroscopies, we study vibrational relaxation of the four cyanide stretching (ν_CN_) vibrations found in [(NH_3_)_5_Ru^III^NCFe^II^(CN)_5_]^−^ (FeRu) dissolved in D_2_O or formamide and [(NC)_5_Fe^II^CNPt^IV^(NH_3_)_4_NCFe^II^(CN)_5_]^4−^ (FePtFe) dissolved in D_2_O. These cyanide-bridged transition metal complexes serve as models for understanding the role high frequency vibrational modes play in metal-to-metal charge transfers over a bridging ligand. However, there is currently little information about vibrational relaxation and dephasing dynamics of the anharmonically coupled ν_CN_ modes in the electronic ground state of these complexes. IR pump-probe experiments reveal that the vibrational lifetimes of the ν_CN_ modes are ∼2 times faster when FeRu is dissolved in D_2_O versus formamide. They also reveal that the vibrational lifetimes of the ν_CN_ modes of FePtFe in D_2_O are almost four times as long as for FeRu in D_2_O. Combined with mode-specific relaxation dynamics measured from the 2D IR experiments, the IR pump-probe experiments also reveal that intramolecular vibrational relaxation is occurring in all three systems on ∼1 ps timescale. Center line slope dynamics, which have been shown to be a measure of the frequency-frequency correlation function, reveal that the radial, axial, and trans ν_CN_ modes exhibit a ∼3 ps timescale for frequency fluctuations. This timescale is attributed to the forming and breaking of hydrogen bonds between each mode and the solvent. The results presented here along with our previous work on FeRu and FePtFe reveal a picture of coupled anharmonic ν_CN_ modes where the spectral diffusion and vibrational relaxation dynamics depend on the spatial localization of the mode on the molecular complex and its specific interaction with the solvent.

## I. INTRODUCTION

Understanding the pathways and timescales of vibrational energy relaxation is crucial for monitoring and controlling chemical reactivity. In polyatomic molecules in solution, vibrational phase and energy transfer and relaxation can occur through intramolecular and intermolecular processes.^1,2^ The vibrational energy transfer processes can be coherent and incoherent in nature and coupled to electronic dynamics in the case of ultrafast photoinduced chemical reactions.^3,4^ Recent experiments on transition metal donor-bridge-acceptor complexes have shown how the vibrations of the bridging species can affect the charge transfer between the donor acceptor moieties.^5–11^ These studies highlight the importance of understanding the time-dependent intra- and intermolecular vibrational energy transfer and relaxation pathways to develop a microscopic understanding of light-induced phenomena in small molecules and materials for efficient photochemical energy conversion.

**FIG. 1.**
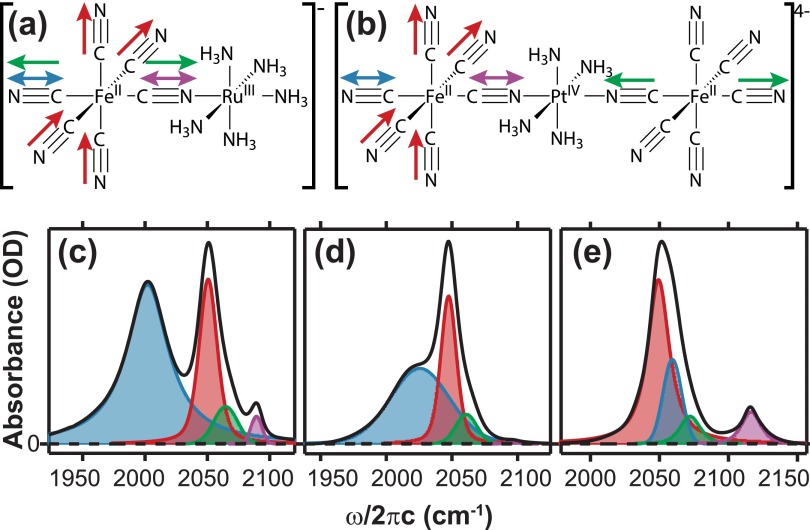
(a) FeRu and (b) FePtFe with their ν_CN_ modes illustrated by colored arrows ν_trans_ (ν_t_, blue), ν_radial_ (ν_r_, red), ν_axial_ (ν_a_, green), and ν_bridge_ (ν_b_, purple). Solvent subtracted FTIR spectra (black line) of (c) FeRu in FA (d) FeRu in D_2_O, and (e) FePtFe in D_2_O. Each FTIR spectrum was fit to four Voigt lineshapes (Ref. 22), and each peak is color coded to match the four ν_CN_ modes shown in panels (a) and (b).

Cyanide-bridged transition metal mixed-valence complexes serve as excellent model systems to study the coupling of vibrational and electronic coordinates in ultrafast charge transfer reactions. Previous ultrafast studies of cyanide-bridged mixed-valence complexes have explored the role played by high-frequency CN stretching (ν_CN_) vibrations in the photo-induced metal-to-metal charge transfer (MMCT) and the subsequent back electron transfer process.^7–9,12–21^ These studies have shown that there is a high degree of vibrational excitation and measured how the vibrational transfer and relaxation pathways evolve as the molecule equilibrates in the ground electronic state following back-electron transfer. However, the intra- and intermolecular vibrational relaxation dynamics of the ν_CN_ modes in the ground electronic state of cyanide bridged transition metal mixed valence complexes have not been explored. The goal of this paper is to determine what role solvent and other molecular properties play in the vibrational relaxation dynamics of the ν_CN_ modes of cyanide-bridged bimetallic and trimetallic transition metal mixed-valence complexes. Infrared pump-probe and two-dimensional infrared (2D IR) spectroscopies are used to measure ground state vibrational dynamics of the cyanide stretching vibrations of [(NH_3_)_5_Ru^III^NCFe^II^(CN)_5_]^−^ (FeRu) dissolved in deuterium oxide (D_2_O) or formamide (FA) and [(NC)_5_Fe^II^CNPt^IV^(NH_3_)_4_NCFe^II^(CN)_5_]^4−^ (FePtFe) dissolved in D_2_O. FeRu and FePtFe both have four cyanide stretching frequencies that are labelled ν_trans_ (ν_t_), ν_radial_ (ν_r_), ν_axial_ (ν_a_), and ν_bridge_ (ν_b_) (illustrated in Figs. 1(a) and 1(b)).

The FTIR spectra of the ν_CN_ modes (see Figs. 1(c)–1(e)) of the bimetallic and trimetallic systems are strikingly different in terms of frequencies, amplitudes, and lineshapes and have been discussed in detail earlier.^22^ The center frequencies of the four ν_CN_ modes for the FeRu complex are arranged in the following ascending order: ν_t_ < ν_r_ < ν_a_ < ν_b_. The frequency ordering changes to ν_r_ < ν_t_ < ν_a_ < ν_b_ for FePtFe dissolved in D_2_O. The higher oxidation state of Pt(IV) compared to Ru(III) explains why the frequency of the bridging ν_CN_ mode is farther to the blue by 20 cm^−1^ for FePtFe than for FeRu dissolved in D_2_O. The larger positive charge pulls more electron density out of the cyanide antibonding (π*) orbital and increases the strength of the CN bond thus increasing the vibrational frequency of the ν_CN_ stretch.^23–25^ If the electron withdrawing effect carries along the MMCT axis, the strength of the CN bond could also increase for the terminal cyanide ligands explaining why the ν_t_ mode (blue arrows in Figs. 1(a) and 1(b)) in FePtFe is at a much higher frequency than it is in FeRu and why the frequency ordering of the four ν_CN_ modes change between the two complexes.

Previous work by our group has measured the experimental 2D IR spectra of FeRu and FePtFe in various solvents at τ_2_ ∼ 100 fs.^22^ The best fits of the 2D IR spectra reveal a picture of a set of weakly coupled anharmonic ν_CN_ modes. The vibrational mode anharmonicities of the individual ν_CN_ modes range from 14 to 28 cm^−1^, and the mixed-mode anharmonicities range from 2 to 14 cm^−1^. Measurements of the relative transition dipole moments of the four ν_CN_ modes reveal that the angles in FeRu in FA are significantly different than both FeRu in D_2_O and FePtFe in D_2_O suggesting that the molecular structure might be a function of solvent environment. In this study, we will explore how the solvent and other molecular parameters affect the intra- and inter-molecular vibrational relaxation and spectral diffusion processes for the ν_CN_ modes in FeRu and FePtFe.

The remainder of this article is organized as follows. Section II describes the methods used to prepare samples and collect both pump-probe and 2D IR spectra. Sections III A and III B describe the results from the IR pump-probe and the 2D IR relaxation experiments, respectively. Section IV contains the discussion of our results followed by a summary in Section V.

## II. METHODS

### A. Materials

Starting materials and solvents were purchased from Sigma Aldrich and used without further purification. The mixed-valence complexes, FeRu and FePtFe, were synthesized as described earlier.^22,26,27^ A saturated solution of FeRu was prepared in FA with a maximum optical density (OD) of 0.22 in the ν_CN_ region after solvent subtraction. A solution of FeRu was also prepared in D_2_O to a concentration of ∼25 mM and maximum OD of 0.36 in the ν_CN_ region. A solution of FePtFe dissolved in D_2_O was prepared to a concentration of ∼15 mM and a maximum OD of 0.45 in the ν_CN_ region for use in experiments.

### B. 2D IR experiments

The experimental parameters and layout of the 2D IR experiments have been described in detail in Ref. 22. Briefly, the 2D IR experiments were performed in a boxcar geometry with ∼80 fs mid-IR pulses centered at 4.9 *μ*m (Δω = 250 cm^−1^). The three input beams were focused to a spot size (1/e^2^) of ∼150 *μ*m at the sample with each beam having energy of 0.4 *μ*J/pulse (0.5 *μ*J/pulse) for the FeRu (FePtFe) samples. The sample was contained in a home built sample cell equipped with two 1 mm thick CaF_2_ windows and a 50 *μ*m Teflon spacer for the D_2_O solutions or a 100 *μ*m Teflon spacer for the FeRu/FA solution. The 2D IR spectra of FeRu in FA were collected at the following τ_2_ delays (waiting times): 0.07, 0.14, 0.225, 0.3, 0.425, 0.475, 0.625, 1, 2.5, 5, 7.5, 10, 15, and 20 ps. The 2D IR spectra of FeRu/D_2_O were collected at τ_2_ delays of 0.09, 0.15, 0.225, 0.3, 0.375, 0.65, 1, 3, 5, 7.5, 10, and 15 ps, and the 2D IR spectra of FePtFe dissolved in D_2_O were taken at τ_2_ delays of 0.15, 0.34, 0.38, 1.36, 2.04, 5, 7.5, 10, and 15 ps. For each sample, the 2D IR spectra were collected in both parallel $\left(S_{∥},\;\text{ZZZZ}\right)$ and crossed $\left(S_{⊥},\;\text{YYZZ}\right)$ polarization geometries. In the former case, all input beams and the signal field have the same polarization and in the latter, beams 1 and 2 have perpendicular polarization relative to beam 3 and the signal field. Magic angle spectra were constructed using the relationship: $S_{M}=\frac{1}{3}\left(S_{∥}+2S_{⊥}\right)$ to remove effects of reorientational dynamics.

### C. IR pump-probe experiments

The generation and manipulation of the mid-IR light pulses used in the IR pump-probe experiments were similar to the 2D IR experiments and has been described earlier.^22,28^ The pump beam (chopped at 500 Hz) and the probe beam were spatially and temporally overlapped in the sample to generate the pump-probe signal. An additional mid-IR pulse was also sent through the sample to act as a reference beam. The input pump and probe beams had energies of ∼0.5 and ∼0.3 *μ*J/pulse for all three samples. All input beams were focused onto the sample with a spot size (1/e^2^) of ∼150 *μ*m. The pump-probe signal and the reference beam were vertically offset before being dispersed by a spectrometer (Triax 190, Horiba Jobin Yvon) and detected on the upper and lower stripes of a 2 × 64 element mercury cadmium telluride array detector (IR0144, Infrared Systems Development).

The pump-probe spectra for the FeRu samples were collected at magic angle where the polarization of the probe beam was rotated 54.7° relative to the polarization of the pump beam to eliminate contributions from reorientational dynamics. Collected data were generated by dividing both the pump on and pump off signals by their respective reference pulses and then subtracting the pump on signal from the pump off signal. Dividing each signal pulse by the reference helped mitigate the effects of shot-to-shot and long term noise fluctuations in the mid-IR field. The reference beam was set to arrive 150 ps before the pump beam to ensure that the sample had relaxed back to the ground state. For FeRu dissolved in FA and D_2_O, the time delay between the pump and probe pulses was scanned from −150 to 150 fs in 10 fs steps, 150 to 2500 fs in 25 fs steps, 2.5 to 5 ps in 50 fs steps, 5 to 8 ps in 250 fs steps, 8 to 20 ps in 1 ps steps, 20 to 40 ps in 4 ps steps, and 40 to 100 ps in 10 ps steps. Each data point represents 1500 shots, and each spectrum was collected 100 times and averaged to obtain the experimental spectra presented here. The experimental spectral resolution is ∼2.8 cm^−1^ for the FeRu samples.

Spectra for FePtFe were collected in both parallel and crossed polarization geometries and no reference beam was used for shot-to-shot normalization of the data. For FePtFe dissolved in D_2_O, the time delay between the pump and probe pulses was scanned from −1.5 to 3.5 ps in 25 fs steps, 3.5 to 6 ps in 250 fs steps, 6 to 20 ps in 1 ps steps, and 20 to 100 ps in 4 ps steps. Each data point represents 2000 shots, and each spectrum was collected 25 times and averaged to obtain the experimental spectra presented here. Experimental spectral resolution is ∼4.4 cm^−1^ for the FePtFe sample.

## III. RESULTS

### A. IR pump-probe experiments

**FIG. 2.**
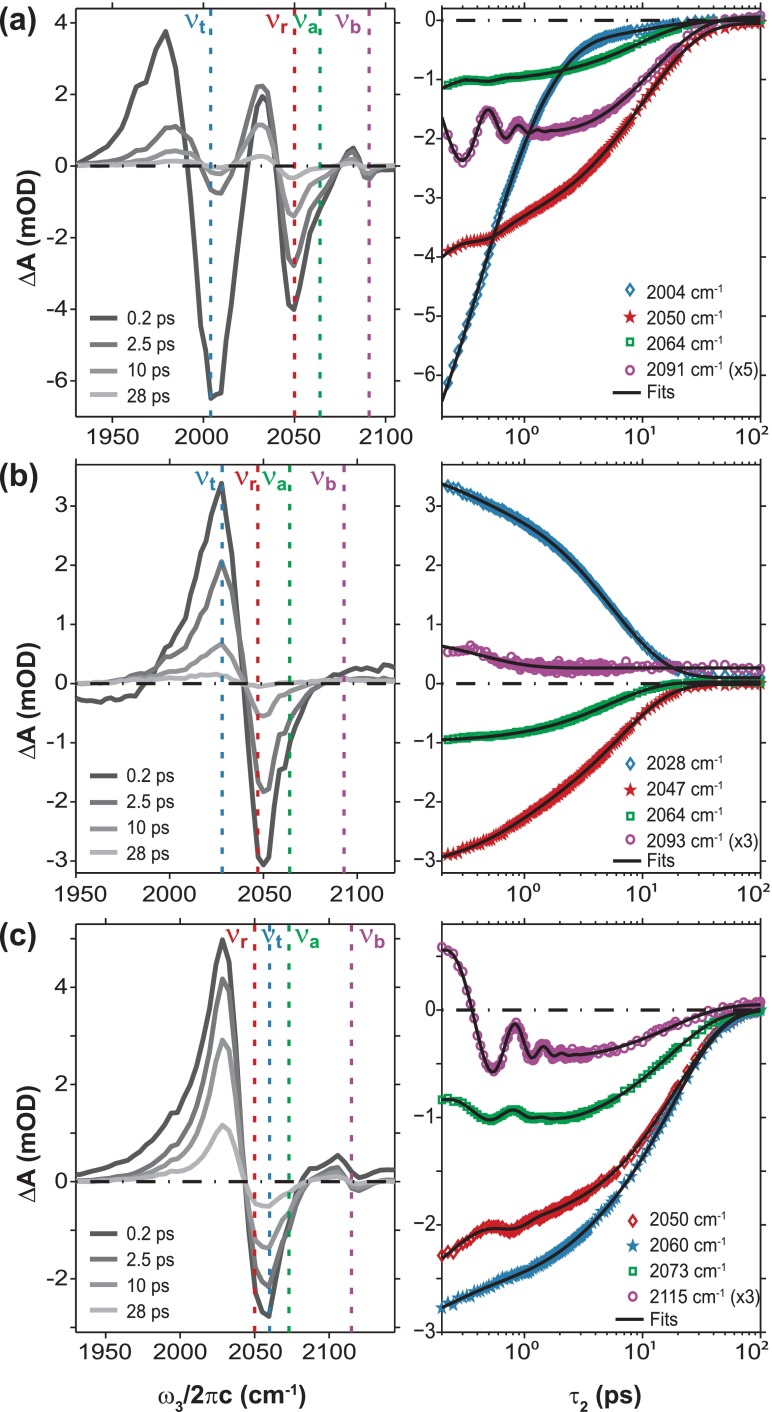
The two panels of (a) represent spectra (left) of FeRu in FA at several τ_2_ delays and time traces (right) of the same signal for selected frequencies shown as dashed lines in the left panel. Note that the time axis follows a logarithmic scale. (b) and (c) The same information for FeRu in D_2_O and FePtFe in D_2_O, respectively. Note that the bridge trace for each sample is multiplied by a static factor to make it easier to compare to the other modes.

The IR pump-probe spectra of FeRu/FA, FeRu/D_2_O, and FePtFe/D_2_O are shown in Figure 2. The time-dependence of the fundamental ν_CN_ frequencies of each sample are shown on semi-log plots. The data were fit to the following expression:

$$\Delta A\left(\tau_{2},\omega_{3}\right)=\left(\sum_{i}A_{i}\;\text{exp}\;\left(-\frac{\tau_{2}}{t_{i}}\right)\right)+A_{rise}\left(1-\text{exp}\;\left(-\frac{\tau_{2}}{t_{rise}}\right)\right)+A_{osc}\;\text{exp}\;\left(-\frac{\tau_{2}}{t_{osc}}\right)\;\text{cos}\;\left(2\pi c\omega_{osc}\tau_{2}+\phi \right),$$ (1)

where *A* and *t* are the amplitude and timescale of each decay/rise, respectively, *ω_osc_* is the frequency of any oscillation that is present, and ϕ is the phase of the oscillation. Because of a large nonresonant signal, we fit the data starting at a pump-probe delay of 200 fs. The best fit parameters are listed in Table I.

**FIG. 3.**
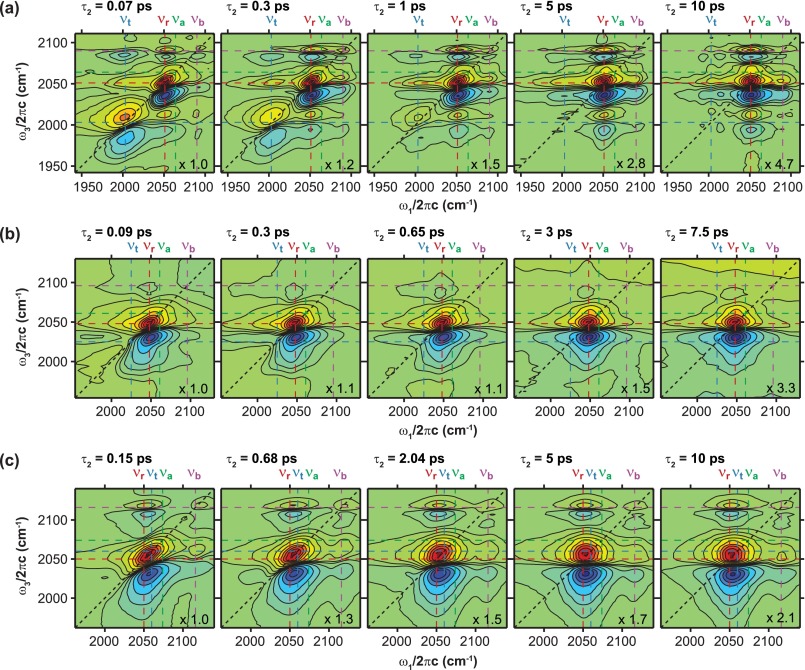
Crossed polarization 2D IR spectra at selected τ_2_ delays for (a) FeRu in FA, (b) FeRu in D_2_O, and (c) FePtFe in D_2_O. All spectra have been normalized to the highest point at each τ_2_ delay, and the relative factors are shown in the bottom right corner. Each FeRu/FA spectrum is plotted using contour lines at the following positions: ±0.018, ±0.055, ±0.1, ±0.2, ±0.35, ±0.5, ±0.65, ±0.8, and ±0.95. Each FeRu/D_2_O spectrum is plotted using contour lines at the following positions: ±0.01, ±0.04, ±0.1, ±0.2, ±0.35, ±0.5, ±0.65, ±0.8, and ±0.95. Each FePtFe spectrum is plotted using contour lines at the following positions: ±0.01, ±0.045, ±0.1, ±0.2, ±0.35, ±0.5, ±0.65, ±0.8, and ±0.95.

Figure 2(a) displays τ_2_ dependent spectrally dispersed pump-probe traces of FeRu/FA in the left panel. The data reveal that the negative bleach signals (0 → 1 transitions) of the ν_r_ (2050 cm^−1^) and ν_a_ (2064 cm^−1^) modes are spectrally overlapped and the weaker axial mode appears as a shoulder to the blue side of the radial mode. It is also evident from Figure 2(a) that the ν_t_ mode (2004 cm^−1^) decays at a much faster rate than the other three modes. The positive absorption signals represent the 1 → 2 transitions to the various overtones and combination bands from the first excited vibrational states. We do not fit the dynamics of the 1 → 2 transitions due to the fact that they have overlapping contributions from both combination bands and overtones and therefore do not represent dynamics of any single transition exclusively. The vibrational relaxation dynamics detected at the four fundamental transitions are plotted in the right panel of Figure 2(a) and the data is fit using Eq. (1). The traces in the right panel of Figure 2(a) and the best fit parameters listed in Table I reveal that the detection of the pump-probe signals at the four fundamental ν_CN_ transition frequencies displays different timescales of vibrational relaxation dynamics. In general, there are two timescales for vibrational relaxation. In all cases, we attribute the short timescale to intramolecular vibrational relaxation and transfer processes where the vibrational energy leaks into other high-frequency ν_CN_ modes or low-frequency vibrational modes along the metal-ligand backbone and the longer timescale to intermolecular vibrational relaxation to the solvent. The signal detected at ω_3_ = ν_t_ decays on two main timescales: 0.31 and 0.99 ps. The fit at ω_3_ = ν_r_ reveals three timescales that consist of two exponential decays and a decaying cosine. We attribute the 10.8 ps decay to vibrational energy relaxation to the solvent. The faster 0.64 ps decay is attributed to cross-population relaxation processes to other intramolecular vibrational modes. The oscillation at ∼50 cm^−1^ results from the coherent superposition of two coupled ν_CN_ modes. The amplitude of the oscillatory component is proportional to the anharmonic coupling and the transition dipole moment between coupled modes. The frequency difference between the radial and trans modes and between the radial and bridge modes is ∼50 and ∼40 cm^−1^, respectively. We have previously measured similar anharmonic couplings and angles between the radial and trans modes and between radial and bridge modes suggesting that the oscillation arises from both of these coherent contributions.^22^ In addition, clear cross-peaks between the ν_t_ and ν_r_ and ν_t_ and ν_b_ modes can be seen in the 2D IR spectra in Figure 3(a) at ω_3_ = ν_t_. The fit of the signal at ω_3_ = ν_a_ (2064 cm^−1^) reveals two timescales consisting of an exponential decay of 9.32 ps and a very weak decaying oscillatory component. The weak oscillatory component at ∼90 cm^−1^ likely results from spectral overlap with the combination bands between the various fundamental ν_CN_ modes. The signal trace at ω_3_ = ν_b_ (2091 cm^−1^) displays an exponential decay of 12.4 ps, a growth at 1.4 ps, and a decaying oscillation at ∼84 cm^−1^. The oscillation matches the difference in frequency between ν_t_ and ν_b_ indicating a superposition between the two modes which have parallel transition dipole moments. In summary, we note that the pump-probe signals detected at the radial, axial, and bridge modes of FeRu/FA display vibrational energy relaxation to the solvent ranging from 9 to 12 ps. The signal detected at the trans mode appears to be an outlier with the longest timescale (with significant amplitude) being ∼1 ps. The intramolecular vibrational transfer and relaxation timescales among the coupled modes range from 0.3 to 1.4 ps and the lifetimes of the coherent oscillations are less than 1 ps.

Figure 2(b) shows the dispersed pump-probe spectra of the FeRu/D_2_O sample as a function of ω_3_ and τ_2_, and the fit values for the fundamental traces can be seen in the FeRu/D_2_O section of Table I. We note that the ν_t_ (2028 cm^−1^), ν_r_ (2047 cm^−1^), and ν_a_ (2064 cm^−1^) modes are spectrally overlapped and the ν_b_ mode (2093 cm^−1^) is extremely weak. In contrast to the pump-probe data for FeRu in FA, the pump-probe traces of FeRu in D_2_O at the chosen frequencies show no rises or oscillations. The fit of the trace at ω_3_ = ν_r_ reveals two timescales, where the short timescale is attributed to intramolecular vibrational relaxation processes, and the 6.51 ps timescale is attributed to vibrational energy relaxation to the solvent. The signal trace at ω_3_ = ν_a_ only decays on a timescale of 5.25 ps. The fit of the signal at ω_3_ = ν_t_ consists of two exponential decays that have amplitude factors of opposite signs to the rest of the fundamental transitions. The fundamental transition for the ν_t_ mode is overlapped with overtones of other modes as well as contributions from combination bands which most likely results in the positive signal. Given that the vibrational relaxation times to the solvent at ω_3_ = ν_r_ and ν_a_ are approximately half of their measured values in the FeRu/FA sample, we tentatively assign the 0.42 ps decay to be indicative of relaxation to the solvent at ω_3_ = ν_t_. Since the ω_3_ = ν_b_ region of the spectrum has a low signal to noise level, fit values for that trace likely have no physical meaning. In summary, we note that the pump-probe signals detected at the radial and axial modes of FeRu/D_2_O have timescales of vibrational energy relaxation to the solvent of ∼6 ps. Interestingly, these timescales are twice as fast as those seen in FeRu/FA.

Figure 2(c) shows τ_2_ dependent dispersed pump-probe spectra of FePtFe and ω_3_ dependent vibrational relaxation dynamics. The ν_r_ (2050 cm^−1^), ν_t_ (2060 cm^−1^), and ν_a_ (2073 cm^−1^) modes are spectrally overlapped, but their individual contributions can be distinguished as shoulders in the strong bleach contribution in the dispersed pump-probe spectra. Similar to the FeRu sample dissolved in FA, we see that pump-probe signal detected at three of the modes in FePtFe have oscillations, but here two modes have visible rises. From the fit values seen in Table I, we can see that the signals detected at all four ν_CN_ modes share a similar long time constant of ∼18 ps representing vibrational energy relaxation to the solvent. The signal detected at the ν_t_ mode does show a faster decay of 0.85 ps, which is attributed to relaxation processes to other intramolecular vibrational modes. The fit for signal detected at the ν_r_ mode also shows two exponential decays representing intramolecular vibrational relaxation (0.5 ps) and vibrational population relaxation to the solvent (19.4 ps). Additionally, the signal at ω_3_ = ν_r_ displays an oscillation with a frequency of 33 cm^−1^. Like seen in the FeRu/FA sample, this oscillation probably arises from contributions of both a ν_r_/ν_t_ superposition, where the difference in frequency is 10 cm^−1^, and a ν_r_/ν_b_ superposition, where the difference in frequency is 67 cm^−1^. Furthermore, a clear cross-peak can be seen between the ν_r_ and ν_b_ modes in the 2D IR spectra in Figure 3(c) at ω_3_ = ν_r_, while the cross-peaks between the ν_t_ and ν_r_ modes are not as easily distinguished due to overlap with the ν_t_ and ν_r_ diagonal peaks. The fit of the signal at ω_3_ = ν_a_ shows one exponential decay representing the vibrational lifetime, a rise, and a decaying oscillation. The rise of ∼1.1 ps is attributed to energy relaxation from the other three ν_CN_ modes into the axial mode. A similar rise of ∼800 fs is seen in the fit of the signal at ω_3_ = ν_b_ (2115 cm^−1^) which also shows one exponential decay and a coherent oscillation. The oscillation frequency of ∼54 cm^−1^ is approximately the difference in frequency between the ν_t_ and ν_b_ modes indicating a superposition between the two modes like that seen in the bridge mode of FeRu/FA. In summary, we note that all four ν_CN_ modes of FePtFe/D_2_O have similar vibrational lifetimes ranging from 16 to 19 ps. The lifetimes of the coherent oscillations found in the pump probe traces detected at the ν_r_, ν_a_, and ν_b_ frequencies are ∼500 fs.

The dispersed pump-probe data for the ν_CN_ modes of FeRu and FePtFe consist of four overlapping vibrations and the fast timescales extracted from the fit to the data probably consist of several contributions arising from vibrational population transfer in and out of various excited modes detected at the particular ω_3_ frequency of interest. A 2D IR relaxation experiment which resolves the excitation frequency axis provides a better description of how vibrational energy transfers between the four coupled ν_CN_ modes and is discussed in detail in Section III B.

### B. 2D IR relaxation experiments

Figure 3 displays representative crossed polarization 2D IR spectra as a function of τ_2_ for all three samples. Note that contour levels do not follow a linear scale and are listed in the figure caption. The 2D IR data for FeRu in FA is shown in Figure 3(a). There are three main features along the diagonal with positive (0 → 1 transitions) and negative (1 → 2 transitions) features. The spectral feature at lowest energy represents the ν_t_ mode, the feature in the middle of the spectrum represents overlapping contributions from the ν_r_ and the ν_a_ modes as well as their cross-peaks, and the one at highest energy represents the ν_b_ mode. It can be seen that the trans mode decays away relatively quickly compared to the other diagonal features. We attribute this to the shorter timescale vibrational relaxation of the ν_t_ mode (1 ps) compared to the other ν_CN_ modes (9–12 ps) as discussed in Section III A and presented in Table I. The time-evolving 2D lineshapes and amplitudes measure the spectral diffusion and vibrational relaxation dynamics of the ν_CN_ modes in FeRu/FA. The 2D peak representing both the radial and axial modes is elongated along the diagonal at τ_2_ = 70 fs and its spectral diffusion dynamics are completed by ∼5 ps. The diagonal and cross-peaks are amplitude modulated as a function of τ_2_ in a 2D IR spectrum of coupled vibrational modes.^29^ Oscillatory behavior is clearly seen for the ν_b_ mode in the 2D IR spectra where the diagonal peak has more amplitude at 300 fs than it does at 70 fs. The beats are also seen in the pump-probe data (Fig. 2(a)) which show a clear oscillation at a frequency of 84 cm^−1^. Recall that the dispersed pump-probe data at a particular τ_2_ delay is the projection of the 2D IR spectrum along ω_3_.^30^ Evidence of intramolecular vibrational energy redistribution among the ν_CN_ modes of FeRu/FA can be observed by the growth of three cross peaks at (ω_1_ = ν_t_, ω_3_ = ν_b_), (ω_1_ = ν_r_, ω_3_ = ν_b_), and (ω_1_ = ν_b_, ω_3_ = ν_r_ /ν_a_) relative to the radial diagonal peak. The time-dependent intensities of the cross-peaks are also plotted in the left panel of Figure 5. The other two obvious cross-peaks at (ω_1_ = ν_r_, ω_3_ = ν_t_) and (ω_1_ = ν_t_, ω_3_ = ν_r_) appear to decay at similar rates to the ν_r_ and ν_a_ modes.

The 2D IR spectra for FeRu in D_2_O are shown in Figure 3(b) and seem very different from the 2D IR spectra of FeRu in FA. The FTIR spectrum of FeRu/D_2_O shows significant overlap of the ν_t_, ν_r_, and ν_a_ modes and very weak amplitude in the ν_b_ mode. The resultant 2D spectra of FeRu in D_2_O show one main peak that represents overlapping contributions from the ν_r_ and ν_a_ modes as well as their cross peaks. At the lower left-hand corner of the central peak, there is a small feature that quickly disappears indicating the ν_t_ mode has an estimated vibrational lifetime of ∼400 fs corresponding to the timescale measured in the pump-probe experiments at ω_3_ = ν_t_ (Table I). A bridge diagonal peak is not distinguishable in any of the spectra due to the ν_b_ mode's very small transition dipole moment ($\left|\mu_{r}\right|=0.89\left|\mu_{t}\right|$, $\left|\mu_{a}\right|=0.40\left|\mu_{t}\right|$, and $\left|\mu_{b}\right|=0.12\left|\mu_{t}\right|$) as diagonal peak amplitudes scale with ${\left|\mu_{p}\right|}^{4}$.^22^ The central diagonal peak loses its spectral correlation and appears fully rounded by ∼5 ps. We note that it is difficult to disentangle the relative homogenous and inhomogeneous contributions from the radial and axial cross peak contributions. There are two cross peaks that show up as wings to the lower and higher energy sides of the central peak (ω_1_ = ν_t_/ν_b_, ω_3_ = ν_r_). The 2D spectra reveal a spectral feature growing in at higher ω_3_ frequencies for τ_2_ > 3 ps. We attribute this to a solvent feature as there is a large D_2_O band centered at ∼2500 cm^−1^. Temperature dependent FTIR spectra of D_2_O show a growth in the transmission from ∼2100 to 2150 cm^−1^ as the temperature increases. In the 2D spectra, we see that the amplitude in the same spectral region increases.

The 2D IR spectra for FePtFe are shown in Figure 3(c). Here, we see two main diagonal features, the central one representing overlapping contributions from the ν_r_, ν_t_, and ν_a_ modes as well as their respective cross peaks, and the higher energy peak representing the ν_b_ mode. The central feature starts off elongated along the diagonal with a similar homogeneous width for all three modes (see 2D IR plot at τ_2_ = 0.15 ps), and the spectral diffusion dynamics are completed by 5 ps. As seen in the 2D spectra for FeRu/FA, the 2D spectral feature of the bridge mode does not appear to have any elongation along the diagonal at early τ_2_ delays. The diagonal bridge peak exhibits amplitude beating with a time-period of 620 fs consistent with the beats seen in the dispersed IR pump-probe data (Fig. 2(c)). We see that the obvious cross peaks (ω_1_ ≈ ν_r_, ω_3_ = ν_b_) and (ω_1_ = ν_b_, ω_3_ ≈ ν_t_) grow in relative to the diagonal features and are most visible in the τ_2_ = 10 ps 2D IR spectrum. Below we will discuss the spectral diffusion and vibrational relaxation dynamics measured in the 2D IR spectra by following the τ_2_-dependent 2D IR line shapes and 2D peak amplitudes, respectively.

The line shapes in the 2D IR experiments are a measure of the transition frequency fluctuations arising from the relative movements of molecules' positions and orientations in solution.^31,32^ Vibrational frequency-frequency correlation functions (FFCFs) can be used to characterize these fluctuations and provide a measure of the system-bath interaction for each vibration. This gives us a tool to look at solvation dynamics in the ground electronic state of molecules in solution. There are various metrics available to extract FFCF timescales, but here we use the center line slope (CLS) method to measure spectral diffusion of the CN stretches.^33^ The CLS method is one way to quantify how elongated the 2D IR peak is along the diagonal at a particular τ_2_ delay. To accomplish this, several ω_1_ frequencies centered on the fundamental frequency of the vibrational mode are chosen, and the ω_3_ frequency at which maximum amplitude is reached is recorded for each ω_1_ frequency. These points determine a line on a ω_1_ versus ω_3_ plot, and the slope of that line is the CLS. This slope is calculated for each τ_2_, and the resulting decay is fit to the following expression to find timescales that are proportional to the FFCF:

$$CLS(\tau_{2},\omega_{3})=\left(\sum_{i}A_{i}\;\text{exp}\left(\frac{-\tau_{2}}{t_{i}}\right)\right)+A_{osc}\;\text{exp}\left(\frac{-\tau_{2}}{t_{osc}}\right)\text{cos}\left(2\pi c\omega_{osc}\tau_{2}+\phi \right)+A_{\infty },$$ (2)

where *A* and *t* are the amplitude and timescale of each decay, respectively, $\omega_{osc}$ is the frequency of the oscillation, and *ϕ* is the phase of the oscillation. The term *A_∞_* represents a static component referring to dynamics that do not occur within the timescale of this experiment. The cosine is only included in the fit if there is an oscillatory component present, and the $t_{osc}$ and $\omega_{osc}$ variables are held constant at the values determined in the IR pump-probe fits at that ω_3_ (see Table I). Previous 2D IR and peak shift experiments have observed oscillations in their extracted FFFCs. Examples include an underdamped hydrogen bond oscillation in HOD/D_2_O,^34^ modulation of the polarization of high frequency NH and OH modes due to strong anharmonic couplings with low frequency skeletal hydrogen bonding modes,^35–37^ and strongly coupled correlated high frequency vibrational modes.^38^

**FIG. 4.**
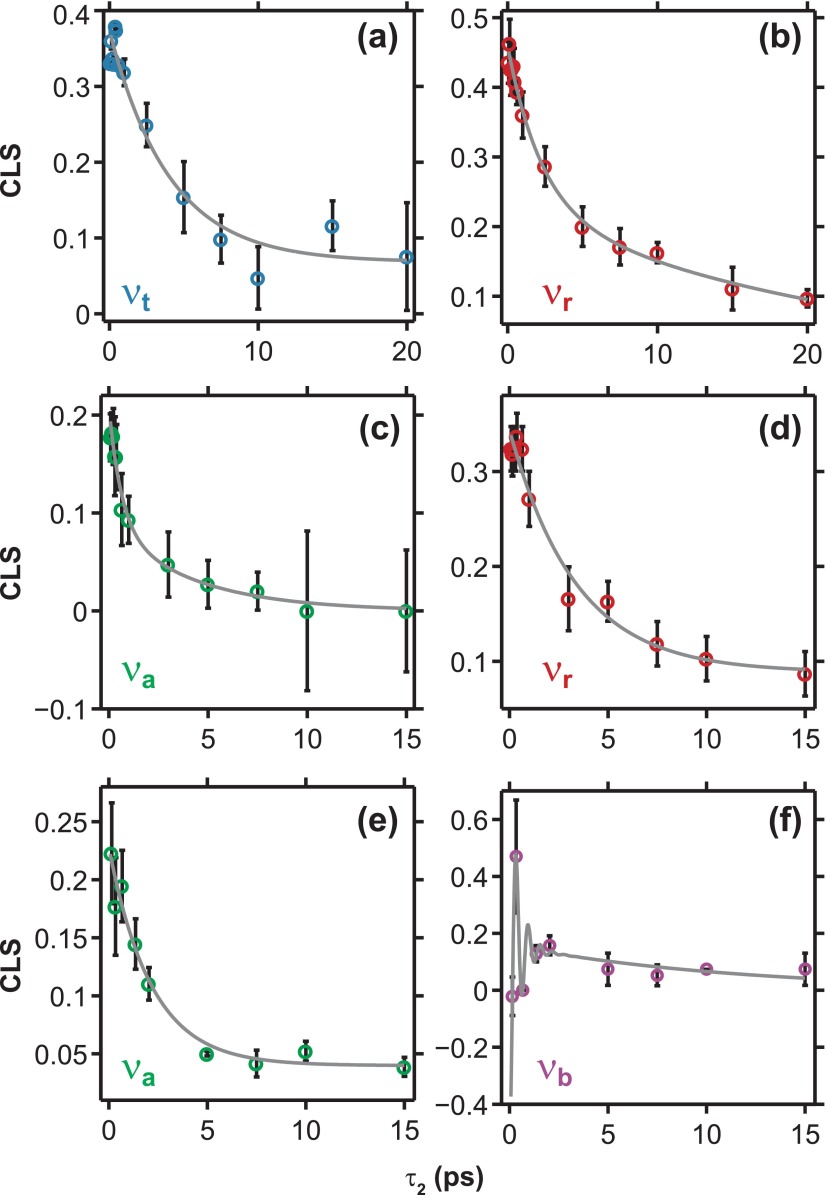
Plots of the center line slope (CLS) measuring spectral diffusion as a function of τ_2_ for selected modes from each sample. Error bars represent 90% confidence intervals on the CLS value for each τ_2_ point. (a) and (b) The ν_t_ and ν_r_ modes, respectively, for FeRu/FA. (c) and (d) The ν_a_ and ν_r_ modes, respectively, for FeRu/D_2_O. (e) (f) The ν_a_ and ν_b_ modes, respectively, for FePtFe.

Figure 4 shows the CLS decays determined from the parallel polarization 2D IR spectra for selected modes from each system and the fits to the experimental CLS traces are listed in Table II. The ν_CN_ modes for which the CLS analysis was performed were chosen carefully to minimize contributions from other close lying modes. Large error bars are due to the limited number of τ_2_ points available for each sample. The CLS of the ν_r_ mode is the only one that can be compared between all three samples. The two CLS traces from the D_2_O samples of both FeRu and FePtFe have a time constant around 3 ps and an offset that represents dynamics too long to be measured with the time resolution of this experiment. The FeRu/FA ν_r_ trace has two time constants and no offset. It still has a short time constant at 2 ps that is close to the D_2_O samples, but its long time constant is ∼20 ps. For the ν_a_ mode, only the two D_2_O samples can be compared. FeRu/D_2_O has two timescales present in its fit: the shortest decay constant measured at ∼600 fs and a longer one at ∼4 ps that is close to the ∼3 ps decays seen in the ν_r_ mode. The FePtFe ν_a_ trace has one time constant at ∼2 ps and an offset. With the ν_b_ mode traces, we cannot compare across molecule or solvent due to low signal in the bridge region of the 2D spectra for FeRu/D_2_O (Fig. 3(b)). The fits for the ν_b_ traces for the FeRu/FA and FePtFe samples reveal a matching decay time constant of ∼20 ps fit to them while keeping the decay for the beat constant. This long decay also matches the long time constant seen in the ν_r_ mode of FeRu/FA. Only the trans mode of the FeRu/FA sample was fully distinguishable from the other modes due to a lack of overlapping spectral features. This allowed a determination of a CLS trace for one ν_t_ mode. Overall, the CLS dynamics reveals that the individual ν_CN_ modes in the mixed valence complexes studied here interact differently with the solvent at a microscopic level resulting in the different timescales of the fits shown in Table II.

**FIG. 5.**
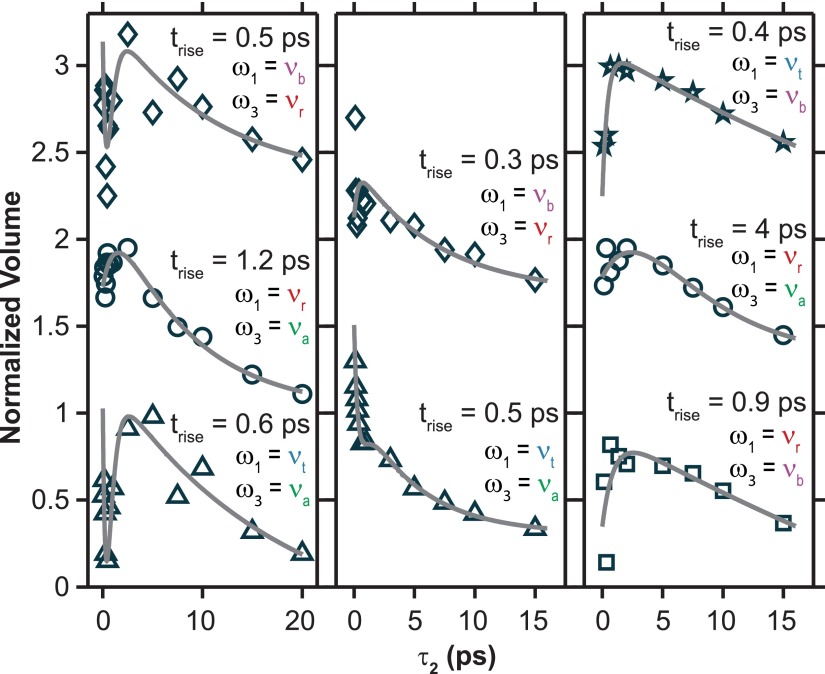
The summed signal intensity over a small area of each 2D IR spectrum centered on the peaks indicated by the ω_1_ and ω_3_ frequencies is shown for each trace for the FeRu/FA samples (left), FeRu/D_2_O sample (middle), and FePtFe sample (right) as a function of τ_2_ delay.

To look at the vibrational dynamics of specific modes using the spectra shown in Figure 3, a small area of each 2D spectrum was chosen for each peak and all signal intensity within that area was summed. These volumes were then plotted as a function of τ_2_ to monitor peak specific relaxation. Each of these decays was then fit to a sum of exponentials like the formula used to fit the pump-probe traces. Because of the low number of τ_2_ points available, these fits have very large uncertainties and only a few fits are shown in Figure 5 for illustration. These fits are discussed in a qualitative manner. Several illuminating time-dependent cross-peak volume plots for FeRu in FA show growths in addition to their decays (Fig. 5 left panel). These rises indicate population transfer between modes on the order of 1 ps. Most of these cross-peaks also have multiple decay timescales with the longest timescales corresponding to the long timescale extracted from the fit of the dispersed pump-probe data at the same ω_3_ frequencies. The mode-specific dynamics of FeRu in D_2_O do not contain as many growths as the FA sample, but the ones that are observed are also on ∼1 ps timescales. Data for FePtFe are similar to that seen for FeRu in FA with multiple rises on the order of 1 ps, several decays, and long timescales on the order of the vibrational lifetimes for the ν_CN_ modes. All of the short decay timescales seen in the pump-probe match the ∼1 ps rises seen in the 2D volume mode-specific relaxation dynamics giving more strength to the argument that intramolecular vibrational transfer dynamics occur in all three systems on a similar timescale.

## IV. DISCUSSION

Our results show that the vibrational relaxation and the vibrational dephasing dynamics of the ν_CN_ modes are mode dependent in the solvated transition metal molecules studied here. Vibrational lifetimes change significantly between modes within the same system and between the same modes in different systems. Long spectral diffusion timescales also show large variations between the same mode in different systems and different modes within the same system. In this section, we will put this work in the larger context of vibrational spectroscopy of CN stretches.

### A. Vibrational relaxation dynamics of the ν_CN_ modes in FeRu and FePtFe

The IR pump-probe experiments reveal that the vibrational lifetimes of the ν_CN_ modes are ∼2 times faster when FeRu is dissolved in D_2_O versus FA. This is likely due to the much larger spectral overlap between the IR bands of the ν_CN_ and D_2_O than exists between the ν_CN_ and FA bands. The D_2_O IR absorption spectrum has a band centered at ∼2500 cm^−1^ which is much broader (∼500 cm^−1^) and more intense than the closest FA band (centered at ∼2210 cm^−1^ with a width of ∼80 cm^−1^) to the ν_CN_ modes studied here. This is particularly obvious in the 2D spectra for the FeRu/D_2_O sample where a large solvent response grows in at high energies along the ω_3_ axis (see Figure 3(b)). A greater overlap of the solvent and solute vibrational spectrum would lead to more efficient energy transfer between the solute and solvent resulting in faster relaxation times as noted previously in experiments of isotope labeled CN ions dissolved in aqueous solutions.^39^ The vibrational lifetime of the trans mode is also likely faster when FeRu is dissolved in D_2_O versus FA, but it is hard to say by how much because of spectral congestion in the FeRu/ D_2_O pump-probe spectra. We note that a faster relaxation time for the ν_t_ mode in D_2_O versus FA was also observed in a similar molecule [(NH_3_)_5_Ru^III^NCOs^II^(CN)_5_]^−^ after optical excitation and back electron transfer.^13^ This was attributed to better overlap between the ν_t_ band and solvent bands in D_2_O compared with FA. Shorter vibrational lifetimes for the ν_t_ mode in FeRu indicate the ν_t_ mode has stronger interactions with the solvent than the other three vibrational modes. The frequency shift of the ν_t_ mode in the FTIR and resonance Raman spectra of FeRu in different solvents provides evidence of the stronger coupling of the terminal CN ligand to the solvent.^15,22^ We also see evidence of strong coupling to the solvent of the ν_t_ mode in recent 2D VE experiments of FeRu/FA.^7^

The IR pump-probe and 2D IR relaxation experiments reveal that the lifetime of the ν_CN_ modes in FePtFe/D_2_O is significantly slower than that of FeRu in FA or D_2_O (see Table I). Due to the larger size and increased complexity of FePtFe, it is expected that the vibrational relaxation for the ν_CN_ modes would be faster than for FeRu. Greater complexity increases the number of intramolecular vibrational relaxation channels available for the ν_CN_ modes to dump their energy into leading to more efficient energy transport out of the ν_CN_ modes. FePtFe also has a larger charge than FeRu and previous work with other CN ions in solution has shown that they relax more quickly with increasing charge, likely due to the increased strength of interaction with the solvent.^40^ In addition to these molecular attributes, most of the FePtFe ν_CN_ modes have better overlap with D_2_O IR bands than the ν_CN_ modes of FeRu. Again, this should increase the efficiency with which energy is funneled out of the ν_CN_ modes and into the solvent. Given these factors, it is surprising to see that the vibrational lifetimes for most FePtFe ν_CN_ modes are almost twice as long as the lifetimes for FeRu/FA and four times as long as the lifetimes for FeRu/D_2_O. The ν_t_ mode certainly decays significantly more slowly in FePtFe than in either FeRu sample. The larger charge on the Pt(IV) compared to the Ru(III) explains why the frequencies of the trans and bridge modes are farther to the blue for FePtFe than for FeRu, as described earlier. The larger positive charge pulls more electron density out of the cyanide antibonding orbital increasing the strength of that bond and increasing its vibrational frequency. This effect might also explain why the characteristics of the trans mode are so different in FePtFe than for the two FeRu systems. If the reduced metal-ligand interaction in FePtFe results in weaker coupling to other carbon-metal modes or other low frequency modes along the MMCT backbone, this would limit interactions with the solvent for FePtFe in general helping explain why the lifetimes observed are so much longer than those measured for the FeRu systems.

Vibrational lifetimes ranging from 2.3 to 38.2 ps were observed for OCN^−^, SCN^−^, and SeCN^−^ in D_2_O,^40^ a lifetime of 170 ps was measured for the ν_CN_ mode in FeNO(CN)_5_^2−^,^41^ and a lifetime of 4.2 ps was observed for a cyanide probe of the villin HP35 protein.^42^ Other simple metal-cyanide compounds have long vibrational lifetimes that range from 8.0 to 170 ps when dissolved in D_2_O.^43^ All of the lifetimes observed in the current study fall within this range with the possible exception of the ∼400 fs lifetime measured for the trans mode in FeRu/D_2_O. Vibrational lifetimes for ν_CN_ modes in FePtFe, all of which are ∼18 ps, closely match the lifetime of 24 ps reported for Fe^II^(CN)_6_^4−^ in D_2_O.^41^ However, the lifetimes for FeRu in D_2_O and FA of ∼6 ps and ∼10 ps, respectively, match the lifetimes reported for Fe^III^(CN)_6_^3−^ in D_2_O and FA of 8 ps and 10 ps, respectively.^41^ At first glance, such a comparison might indicate that the oxidation state of the iron in FeRu might be +3 instead of the +2 reported or at least more delocalized than assumed. However, work by multiple groups has shown that the extra electron is fairly localized on the Ru and not on the Fe.^44,45^ In a study of single metal-cyanides of the form M(CN)_x_^y−^ dissolved in D_2_O, it was found that the vibrational lifetimes for the ν_CN_ mode was split into two camps where the first group have long vibrational energy relaxation times of >100 ps, and the second group have times of <35 ps. The first group contains Pt(CN)_4_^2−^, a square planar molecule, while the second includes Ru(CN)_6_^4−^, Fe(CN)_6_^2−^, and Fe(CN)_6_^3−^, all octahedral molecules.^43^ While the oxidation state and molecular configuration of the platinum investigated in this study are different than the state of the platinum in FePtFe, the large discrepancy in relaxation rates between the Pt and the Fe containing species, despite the species with similar charges, might provide a clue as to why we observe longer lifetimes for the ν_CN_ modes in FePtFe than in the ν_CN_ modes in FeRu.

### B. Spectral diffusion dynamics of the ν_CN_ modes in FeRu and FePtFe

Due to the limited number of points available to fit and the large error bars that result for the CLS timescales measured, the comparisons that can be made between the samples studied here and other systems are limited. Approximately 3 ps timescales seen repeatedly in the CLS measurements can be compared with similar timescales of 1–2 ps seen in the frequency-frequency correlation functions of vibrational probes in several compounds: a 1.5 ps spectral diffusion constant for the ν_CN_ mode in Fe^II^(CN)_6_^4−^ in D_2_O,^46,47^ a ∼2 ps timescale for ν_NO_ mode FeNO(CN)_5_^2−^ in several hydrogen bonding solvents,^48^ and a 1.6 ps timescale for the cyanide probe of a villin HP35 protein when water molecules were allowed into the pocket.^42^ In all of these cases, the timescale was compared to the characteristic timescale for the forming and breaking of hydrogen bonds.^34,49^ In light of this, the lack of a ∼3 ps timescale for the bridge mode CLS could be attributed to its greater isolation from the solvent. The bridging cyanide is less likely to form hydrogen bonds with the solvent because the nitrogen is already bound to the ruthenium instead of being free to interact with the solvent like it is for the other CN ligands. Comparing the ν_r_ traces between all three samples, there is a dependence on solvent in that both D_2_O samples only have one decay and an offset, whereas the FA sample has a similar short decay and a longer decay with no offset. This would suggest that, at least for this mode, spectral diffusion rates could rely on the structure of the molecule. But we can see a difference in the spectral diffusion timescales between the molecules by comparing the ν_a_ traces between FeRu and FePtFe in D_2_O. The FePtFe sample only shows the ∼1 ps decay and an offset. In contrast, the FeRu sample shows a shorter timescale, the ∼1 ps decay, and no offset. The lineshape analysis of the CN modes in FeRu and FePtFe reveal a complex picture of solute-solvent interactions in these transition metal mixed-valence complexes. This complex picture is also borne out by the X-ray absorption spectroscopy of FeRu dissolved in water at the Ru L_3_ edge which has revealed that the explicit hydrogen bonding environment of the solvent is essential to stabilize the complex with the correct oxidation states of the transition metal atoms.^45^

## V. SUMMARY

The goal of this study was to investigate the vibrational relaxation and dephasing dynamics for a set of four anharmonically coupled cyanide stretching vibrations of bimetallic and trimetallic transition metal mixed-valence complexes. We measure vibrational lifetimes, intramolecular vibrational transfer dynamics, and spectral diffusion dynamics using infrared pump-probe and two-dimensional infrared spectroscopies. The values for vibration lifetimes reveal that the ∼18 ps lifetimes for the ν_CN_ modes of FePtFe dissolved in D_2_O are approximately four times longer than the modes of FeRu dissolved in D_2_O and two times longer than the modes in FeRu dissolved in FA. Vibrational lifetimes for the trans mode in both FeRu systems are significantly faster than the other three modes, which is not the case for FePtFe. This is an indication that FePtFe has weaker solute-solvent interactions since the trans mode is the most accessible to the solvent. Mode specific dynamics from both the pump-probe and 2D IR experiments reveal that intramolecular vibrational transfer processes are occurring in all three systems on the order of 1 ps. Spectral diffusion dynamics reveal that the radial, axial, and trans ν_CN_ modes show a ∼3 ps timescale that is indicative of the forming and breaking of hydrogen bonds between the solvent and each ν_CN_ mode that is not apparent in the bridge mode. These results along with our previous work on these complexes reveal a complex solute-solvent environment with coupled anharmonic ν_CN_ modes where the spectral diffusion and vibrational relaxation dynamics are different for specific modes depending on their spatial localization on the molecular complex. Recent 2D VE results clearly indicate that only some of the ν_CN_ modes are coherently coupled to the metal-to-metal charge transfer transition. The vibronic nature of the ν_CN_ modes adds an additional layer of complexity to analyzing the vibrational relaxation processes in the ground electronic state. The results in this work present a challenge to theorists interested in calculating structural dynamics of charge transfer systems where the electronic, vibrational, and solvent degrees of freedom are intrinsically coupled.

## Figures and Tables

**TABLE I.** Parameters from the exponential fits to experimental pump-probe data. Error bars represent 95% confidence intervals.

	Mode	Freq (cm^−1^)	*t_1_* (ps) (*A_1_*)	*t_2_* (ps) (*A_2_*)	*t_3_* (ps) (*A_3_*)	*t_rise_* (ps) (*A_rise_*)	*t_osc_* (ps) (*A_osc_*)	*ω_osc_* (cm^−1^)
FeRu/FA	Trans	2004	0.31 ± 0.02 (−0.52)	0.99 ± 0.04 (−0.44)	10. ± 1 (−0.04)	…	…	…
	Radial	2050	0.64 ± 0.04 (−0.18)	10.8 ± 0.1 (−0.77)	…	…	0.2 ± 0.1 (−0.05)	50 ± 10
	Axial	2064	9.32 ± 0.09 (−0.97)	…	…	…	0.7 ± 0.3 (−0.03)	90. ± 4
	Bridge	2091	12.4 ± 0.5 (−0.51)	…	…	1.4 ± 0.2 (−0.14)	0.30 ± 0.02 (0.35)	84 ± 1
FeRu/D_2_O	Trans	2028	0.42 ± 0.03 (0.15)	5.81 ± 0.05 (0.85)	…	…	…	…
	Radial	2047	0.72 ± 0.03 (−0.24)	6.51 ± 0.09 (−0.76)	…	…	…	…
	Axial	2064	5.25 ± 0.06 (−1)	…	…	…	…	…
	Bridge	2093	0.41 ± 0.06 (1)	…	…	…	…	…
FePtFe	Trans	2060	0.85 ± 0.07 (−0.12)	17.2 ± 0.2 (−0.88)	…	…	…	…
	Radial	2050	0.5 ± 0.1 (−0.13)	19.4 ± 0.2 (−0.76)	…	…	0.34 ± 0.06 (−0.11)	33 ± 4
	Axial	2073	16.1 ± 0.3 (−0.71)	…	…	1.11 ± 0.09 (−0.20)	0.57 ± 0.08 (−0.09)	52 ± 2
	Bridge	2115	18 ± 1 (−0.26)	…	…	0.82 ± 0.08 (−0.23)	0.46 ± 0.02 (−0.51)	53.6 ± 0.6

**TABLE II.** Exponential fit values to CLS traces. Error bars represent 90% confidence intervals.

	Mode	t_1_ (ps) (A_1_)	t_2_ (ps) (A_2_)	ω_osc_ (cm^−1^)^a^	A_∞_
FeRu/FA	Trans	4 ± 2 (0.31)	…	…	0.07 ± 0.05
	Radial	2 ± 1 (0.23)	20 ± 10 (0.23)	…	…
	Bridge	20 ± 40 (0.21)	0.30 (0.3)	84	…
FeRu/D_2_O	Radial	3 ± 1 (0.26)	…	…	0.09 ± 0.03
	Axial	4 ± 4 (0.09)	0.6 ± 0.7 (0.12)	…	…
FePtFe	Radial	3 ± 3 (0.83)	…	…	0.1 ± 0.3
	Axial	2 ± 1 (0.19)	…	…	0.04 ± 0.02
	Bridge	11 ± 9 (0.16)	0.46 (0.7)	54	…

^a^ Oscillation frequencies are held constant at their respective pump-probe oscillation frequency.
